# Zinc (Zn): The Last Nutrient in the Alphabet and Shedding Light on Zn Efficiency for the Future of Crop Production under Suboptimal Zn

**DOI:** 10.3390/plants9111471

**Published:** 2020-10-31

**Authors:** Gokhan Hacisalihoglu

**Affiliations:** Department of Biological Sciences, Florida A&M University, Tallahassee, FL 32307, USA; gokhan.h@famu.edu

**Keywords:** zinc, sustainability, food security, seed quality, zinc efficiency, staple foods, crops

## Abstract

At a global scale, about three billion people have inadequate zinc (Zn) and iron (Fe) nutrition and 500,000 children lose their lives due to this. In recent years, the interest in adopting healthy diets drew increased attention to mineral nutrients, including Zn. Zn is an essential micronutrient for plant growth and development that is involved in several processes, like acting as a cofactor for hundreds of enzymes, chlorophyll biosynthesis, gene expression, signal transduction, and plant defense systems. Many agricultural soils are unable to supply the Zn needs of crop plants, making Zn deficiency a widespread nutritional disorder, particularly in calcareous (pH > 7) soils worldwide. Plant Zn efficiency involves Zn uptake, transport, and utilization; plants with high Zn efficiency display high yield and significant growth under low Zn supply and offer a promising and sustainable solution for the production of many crops, such as rice, beans, wheat, soybeans, and maize. The goal of this review is to report the current knowledge on key Zn efficiency traits including root system uptake, Zn transporters, and shoot Zn utilization. These mechanisms will be valuable for increasing the Zn efficiency of crops and food Zn contents to meet global needs for food production and nutrition in the 21st century. Furthermore, future research will address the target genes underlying Zn efficiency and the optimization of Zn efficiency phenotyping for the development of Zn-efficient crop varieties for more sustainable crop production under suboptimal Zn regimes, as well food security of the future.

## 1. Introduction

**Figure 1:**
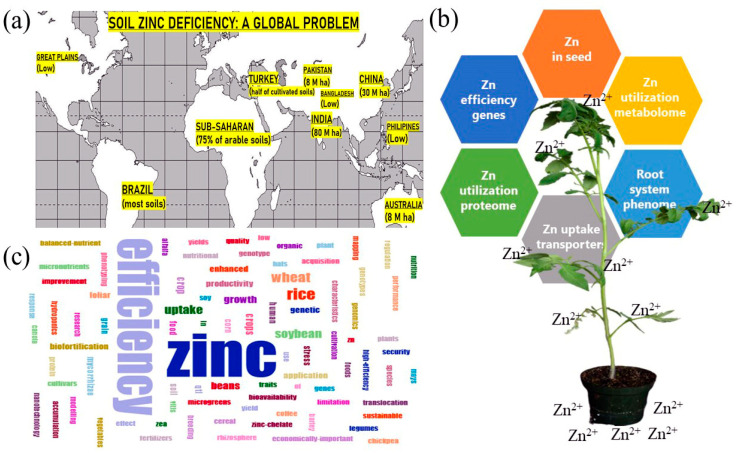
Overview of Zn deficiency and plant Zn efficiency. (**a**) Zn deficiency world map showing major countries and regions with low-Zn soils; (**b**) potential plant Zn efficiency approaches; and (**c**) word cloud of plant Zn efficiency keywords from the literature.

It is estimated that global food crop production must double in order to feed the increased world population of 10 billion by the year 2050 [1]. Zinc (Zn) deficiency, together with vitamin A and iron (Fe) deficiencies, are the most common nutritional disorders, especially in developing countries [2]. Research shows that 17.3% of people worldwide are at Zn deficiency risk [3]. Zn is one of the 17 essential mineral nutrients and plays an important role in plant growth, function, gene expression, structures of enzymes, photosynthesis, pollen development, sugar transformation, protein synthesis, membrane permeability, signal transduction, and auxin metabolism [4,5,6]. Plants take up the Zn from the soil and soil Zn deficiency has become a critically important abiotic stress factor, affecting over 49% of arable lands worldwide (Figure 1a) [5,6,7,8]. Zn deficiency negatively affects plant growth, causing stunting short internodes, small leaves, and interveinal leaf chlorosis, as well as delayed maturity and necrotic tissue death in severe cases [4]; therefore, adequate Zn is essential for crop yield and quality. Moreover, the use of synthetic fertilizers is often insufficient to alleviate soil Zn deficiency.

In order to reduce Zn deficiency throughout the susceptible regions, research has been conducted in various countries that are low in Zn, such as Turkey, Australia, Brazil, India, and China [8,9,10,11]. Plants with high Zn efficiency exhibit high yield and significant growth under low Zn supply [9]. Identifying, developing, and growing Zn-efficient crop varieties could provide approaches for managing low-Zn stress in soils to minimize yield and quality losses [4]. Moreover, elucidating the mechanisms of Zn efficiency will provide important information for improving crop nutrition, as well as sustainable global food systems [4,8,11].

Zn is also essential for human nutrition and development, therefore highlighting the importance of improved Zn contents in staple food crops such as rice, wheat, maize, beans, and others [12]. Understanding the mechanisms of Zn transport and distribution within crops could inform efforts to improve the Zn content of key foods. For example, an effective approach in recent years is biofortification (biological fortification), enriching crops using transgenic techniques, agronomic practices, or conventional crop breeding, which offers sufficient levels of Zn via cereals, vegetables, beans, and fruits to the targeted regions worldwide [13].

This review will focus on advances in the strategies of how crop plants respond to low Zn availability to cope with low-Zn stress conditions, as well as current knowledge of Zn efficiency and future research directions.

## 2. Soil Zn Deficiency

Zn, a divalent cation, was established as an essential micronutrient for higher green plants in 1926 by Sommer and Lipman [14]. The type of soils affected by Zn deficiency include all soils with low Zn availability, such as high pH calcareous soils, intensively cropped soils, sandy soils, and high P soils [15]. About half of soils are naturally low in Zn worldwide [5]. When it comes to low-Zn soils, there are many countries with soils extensively deficient in Zn [4,5]. For example, Zn is mostly deficient in the majority of soils in Bangladesh, Brazil, Pakistan, the Philippines, and Sudan. Furthermore, Zn is deficient in approximately 75% of the arable soils in sub-Saharan Africa, 50% of the cultivated soils in India, 50% of the cultivated soils in Turkey, 45% of soils in western Australia, and 33% of the soils in China (Figure 1) [4,5]. It has been reported that there is Zn deficiency in the Great Plains and western regions of the United States [16] and sandy soils in Florida [17]. It appears that the use of synthetic fertilizers is not necessarily sufficient for alleviating sub-soil Zn deficiency. Therefore, identifying, developing, and growing Zn-efficient crop varieties are preferred ways to manage low-Zn stress in soils to minimize yield and quality losses [4,9]. Hundreds of genotypes of wheat (*Triticum aestivum*) [8], beans (*Phaseolus vulgaris*) [9], chickpeas (*Cicer arietinum*) [18,19], and rice (*Oryza sativa*) [20,21] were screened for Zn efficiency to accomplish this goal. Plant Zn efficiency screening refers to both visual symptom rating systems as well as biomass and yield under low and sufficient Zn conditions [9,10]. More recent high-throughput phenotyping systems will be beneficial for improving plant Zn efficiency assessment and prediction (Figure 1b,c). The development of cereal or vegetable cultivars with higher Zn efficiencies suitable for low-Zn soils is important for sustainable agricultural production and reduced fertilizer input, as well as population growth. Furthermore, the availability of Zn-efficient cultivars will increase the cultivation of them worldwide.

## 3. Evidence of Natural Genetic Variation for Plant Zn Efficiency: A Large Untapped Resource for Overcoming Low-Zn Stress

**Figure 2:**
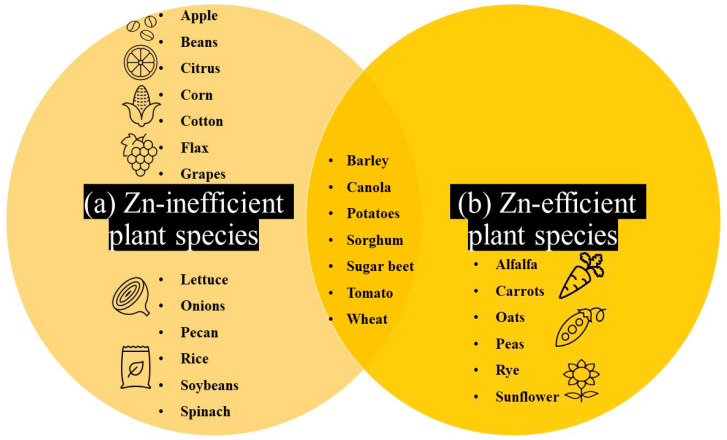
Venn diagram showing the Zn-inefficient plant species (**a**) and Zn-efficient plant species (**b**); overlap area: mildly Zn-efficient plant species.

Soil Zn deficiency can cause negative impacts on yield and therefore economic losses [7]. One key approach for crop improvement is identifying beneficial natural alleles and using association studies to reveal the mechanisms underlying natural variation in Zn efficiency. Therefore, exploring natural variation can be beneficial for crop breeding and selection. Indeed, many crop species and varieties show considerable variation in Zn efficiency. While plant species such as alfalfa, carrots, oats, peas, rye, and sunflower are considered Zn efficient, apples, beans, citrus, cotton, flax, grapes, lettuce, onions, pecans, rice, soybeans, spinach, and sweet corn are considered Zn inefficient. Moreover, plant species such as barley, canola, potatoes, sorghum, sugar beet, tomato, and wheat display medium-level Zn efficiency (Figure 2a,b) [3,5].

It is well known that if researchers can identify crop traits that improve Zn efficiency, growers could have improved yields in Zn-poor soils worldwide. Significant genotypic differences for Zn efficiency have been observed in many crop species, such as rice (*Oryza* sativa) [20.21], wheat (*Triticum aestivum*) [9], common beans (*Phaseolus vulgaris*) [10], maize (*Zea mays*) [22], sorghum (*Sorghum bicolor*) [23], soybeans (*Glycine max*), tomatoes (*Solanum lycopersicum*) [24], chickpeas (*Cicer arietinum)* [18,19], barley (*Hordeum vulgare*) [25], and pigeon peas (*Cajanus cajan*) [26]. There is increasing importance for Zn-efficient cultivars that could adapt to and cope with low-Zn soils. Moreover, while the above list is not exhaustive, there are certain staple crop species with a broad screening of a large number of genotypes in low-Zn soil [27,28]. For the past two decades, there has been substantial research into the Zn efficiency of wheat, beans, and rice. Taking wheat (*Triticum aestivum*) as an example, several studies have shown that wheat genotypes differ widely in their Zn efficiency when grown in low-Zn alkali soils of Central Turkey [29], southern Australia [27], China [30], and Brazil [31]. As a result, there are few Zn-efficient genotypes identified based on extensive field studies [29,32].

Common bean (*Phaseolus vulgaris*) is a prevalent, protein-rich legume crop that is extremely sensitive to low-Zn stress in soil (Figure 2a). A large number of screenings of common bean genotypes in Zn-deficient soil experiments have identified the most Zn-efficient genotypes [10]. Blair et al. [33] investigated Zn accumulation in common beans, utilizing low-mineral (DOR364) and high-mineral (G19833) genotypes and identified the linkage group B11 as an important locus for the Zn efficiency trait.

Rice (*Oryza sativa*) is one of the most important staple food crops for humans and feeds over half of the world population. In the U.S. alone, rice is an economically important commodity with a yearly economic value of USD 3 billion [34]. Zn deficiency in rice was first reported in the 1960s in the U.S. [35]. Furthermore, rice is mainly cultivated in soils with low Zn availability (Figure 1a and Figure 2a). Recent studies showed have revealed that there is a wide genetic variation in Zn efficiency in rice, and Zn in seeds is negatively correlated with yield [21]. Recently developed high-throughput phenotyping systems will improve the assessment and prediction of Zn efficiency.

Maize (*Zea mays*) is the third most important cereal crop globally and the first crop with reported Zn deficiency symptoms [36]. It was reported that there is significant genotypic variation among maize cultivars in Brazil [37].

## 4. Zn Efficiency Strategies in Crop Plants

Zn is a critical nutrient for plants [4] and certain plant species and varieties have developed strategies for securing an adequate supply or maximizing utilization of Zn. Zn-efficient crops and plant varieties are able to achieve sustainable growth and production as well as yield, especially in alkali soils, and could therefore be used to address the Zn deficiency problem. However, it is necessary to better understand the mechanisms of Zn efficiency, as well as natural variation in Zn efficiency traits in food crop plants. Although natural variation in Zn efficiency has been extensively studied in wheat, beans, rice, and chickpeas, the underlying physiological and genetic mechanisms are still not well understood [4,5,6,7]. Zn efficiency is a complex trait with two major mechanisms at a number of levels (Figure 1b). Furthermore, Zn efficiency could be explained with other potential mechanisms, as well as the combined effect of more than one mechanism.

### 4.1. Plant Zn efficiency Mechanism Candidate 1—Zn Uptake Systems and Transporters of Zn

In the uptake process, Zn^2+^ ions travel through the root epidermis, cortex, endodermis, pericycle, and xylem and are then translocated to the stem, leaves, phloem, and seeds [38]. In the past three decades, many attempts have been made to reveal the mechanisms of Zn-efficient plants in response to low-Zn stress in order to determine effective crop breeding strategies [39]. There have been various Zn efficiency mechanisms proposed for food crops in the literature; however, considerable experimental evidence comes from root uptake studies [4,9,15,39,40]. A number of recent uptake studies in crop plants found no strong correlation between root Zn^2+^ influx and Zn efficiency, especially in wheat [6,11,40]. This indicates that Zn efficiency in higher plants is likely not a root-focused trait but a shoot-focused trait. Furthermore, this was supported by the findings that the availability of Zn in soil solution may be an important cause of low-Zn stress compared to total Zn in the soil [15].

Zn uptake can be facilitated by root hairs that increase the availability of Zn from the soil [2]. It is well known that soil type and pH are important determinants of how much Zn is available for crop plants to use [2,9]. Soil pH is important for Zn because it can form insoluble complexes, especially in alkaline (high pH and high CaCO_3_) soils [41]. Zn deficiency is also common in sandy soils with low total Zn availability [2]. Furthermore, biological factors such as phytosiderophores could affect Zn availability by exudation. As an example, Rengel and Graham [42] found that Zn deficiency caused Fe deficiency may be the major factor that leads to phytosiderophore release by Zn-efficient wheat varieties.

The uptake of Zn into the root follows a biphasic pattern of the high affinity transport system (HATS) and low affinity transport system (LATS) before remobilization [4]. While the LATS mechanism functions when Zn is at high concentrations, the HATS mechanism functions at low external Zn concentrations [25,43]. In wheat, our previous studies demonstrated that Km values were 0.6 to 2nM for HATS and 2 to 5 µM for LATS [4,25]. Milner et al. [43] further suggested a widespread role of the high affinity pathway within plants.

Zn is transported across the root plasma membrane into root cells by transporter proteins such as ZRT-IRT-like protein (ZIP) family, HMA (heavy metal ATPase) family (P-type ATPase), MTP (metal tolerance protein) family cation diffusion facilitators (CDFs), vacuolar iron transporter (VIT) family, and plant cadmium resistance family (PCR) proteins [44,45]. There are transporter genes such as NAS2, NAS4, ZIP4, and IRT3 that act as free Zn^2+^ sensors in the Arabidopsis genome [46]. There are Zn transporters such as MTP and HMA that are affected by Zn deficiency [47]. Zn transporter genes have been shown to have their expression regulated by transcription factors, such as bZIP19 and bZIP3, depending on cytoplasmic free Zn changes [48]. Other transporters involved in Zn uptake include OsHMA2 (in pericycle), OsZIP9, and OsZIP7 (in xylem) [49]. Additionally, it was hypothesized that phytosiderophores, which are organic substances produced by plants, including nicotinamine, mugeniec acid, and avenic acid, may promote Zn uptake, especially in rice in waterlogged soils with high Fe and low Zn levels [50]. Other Zn transporter families included P-type ATPase (metal transporting ATPases), cation diffusion facilitators (CDFs), CAX (cation exchangers) proteins, and natural resistance-associated macrophage protein (NRAMP) [38]. Future research on the characterization of Zn transporter proteins will help to understand how crop plants tolerate low-Zn soils.

### 4.2. Plant Zn Efficiency Mechanism Candidate 2—Shoot Internal Zn Utilization

Zn is regarded as the only metal that is involved in all enzyme classes, including lyases, transferases, isomerases, oxidoreductases, and hydrolases [4], which subsequently may affect Zn efficiency. Moreover, it was reported that Zn deficiency caused the inhibition of carbonic anhydrase in crop plants [4,15]. Therefore, it is required for the efficient functioning of more than 300 enzymes [4,5]. The use of more Zn-efficient crops will help to maintain crop yields in the future. It has been suggested that Zn efficiency points to the existence of a shoot-coordinated pathway [32,51,52]. One of the complex Zn efficiency mechanisms is the internal biochemical utilization of Zn in the shoot system. Considering the fact that Zn-efficient and Zn-inefficient crop plants have similar leaf Zn concentrations, Zn-efficient varieties have to be using their greater internal utilization efficiency mechanisms. There are several key enzymes that require Zn as part of their essential components [9]. As a result, considerable recent experimental evidence has been presented that plant shoot internal Zn utilization is based on enzymes requiring Zn [4,5,32,53,54]. It has been proposed that greater activities of carbonic anhydrase and Cu/Zn superoxide dismutase enzymes may be responsible for the increased utilization of cytoplasmic Zn in Zn-efficient wheat genotypes compared with inefficient genotypes [53]. Finally, this was further supported by higher expression of the genes for Zn-requiring enzymes, including Cu/Zn superoxide dismutase [4,51]. A study was carried out with wheat that reported that physiological Zn utilization plays an important role in Zn efficiency and grain Zn concentration was correlated with superoxide dismutase and carbonic anhydrase activities [55]. Additional future research will help further our understanding of Zn efficiency by discovering novel genes on shoot internal Zn utilization with regard to Zn enzymes in crop plants.

### 4.3. Other Mechanisms

Additional Zn efficiency mechanisms may be operating in crop plants (e.g., root system architecture or seed Zn) and future studies are needed to identify and characterize these [56]. Furthermore, it has been reported that soil conditions, together with the environmental conditions of geographic locations, can impact micronutrient contents, such as Zn in seeds [4,8,33]. For example, Zn concentration in plant parts such as seeds is an important parameter for human nutrition. Previous research reported seed Zn content QTLs (quantitative trait loci) in wheat [57], rice [58], maize [59], and beans [60] that can be used in the marker-assisted selection and breeding of Zn-biofortified crop varieties. There are 22 QTLs of concentration of Zn, copper (Cu), and cadmium (Cd) identified in brown rice [61]. There are two major QTLs of Zn efficiency and seed Zn accumulation identified in wheat [62]. Moreover, there are grain Zn and iron (Fe) QTLs on chromosome 1, 4, 7, and 11 in rice [63]. This will increase our understanding of plant Zn efficiency physiology and molecular genetics and contribute greatly to improving crop tolerance to low-Zn soils around the world.

## 5. Conclusions, Future Challenges, and Perspectives

Zn impacts not only plant growth and function but also human nutrition since plants are a dominant part of diets. Our understanding of the impact of Zn in living organisms continues to advance in Zn-efficient crop varieties that can cope with low-Zn stress in soils. A comprehensive understanding of plant Zn efficiency strategies, cellular mechanisms, and genes can facilitate opportunities for increasing agricultural sustainability, improving human nutrition, and reducing synthetic fertilizer usage. In turn, Zn efficiency could enhance crop production and nutritional quality for the increasing population of the 21st century.

There is a need for more research and some of the suggested research approaches to further explore Zn efficiency may include the following: (1) Identifying the target genes and pathways for Zn efficiency in plants; (2) investigating potential genome editing technologies (CRISPR-Cas9) [64]; (3) developing new methods to advance Zn efficiency phenotyping for food crops in the field; (4) metabolomic profiling of Zn efficiency responses under low-Zn stress in crop plants; and (5) genome-wide association studies (GWASs) to detect the genetic basis of Zn efficiency and seed Zn accumulation under low Zn stress environments.
